# Supplementary material for: Patterns of Intron Gain and Loss in Fungi
Source: PLoS Biol. 2004 Nov 30;2(12):e422. doi: 10.1371/journal.pbio.0020422 (PMC532390; doi:10.1371/journal.pbio.0020422)
Supplement: Table S1 — Also available at http://genes.mit.edu/NielsenEtAl/. (4.3 MB ZIP). [file pbio.0020422.st001.zip › NielsenEtAl/html/92.html]

AN4492.1.NCU00823.1.MG05858.1.FG00781.1


```
 CLUSTAL W (1.82) Multiple Sequence Alignments - Introns Inserted


Sequence 1: NCU00823.1	420 aa
Sequence 2: FG00781.1	337 aa
Sequence 3: MG05858.1	335 aa
Sequence 4: AN4492.1	344 aa
Alignment Length: 424 aa
Number Identitical Residues: 243 aa
Alignment Score (without introns) 10037


MG05858.1 	--------------------------------~---------------------------
NCU00823.1	MVFSSSSVPDAGVSVTEWQNCLYEPRHNAEVA1QGNLPSPGWPAVDFVAVGVAGCHSLSS
FG00781.1 	--------------------------------~---------------------------
AN4492.1  	--------------------------------~---------------------------
          	                                                            

MG05858.1 	-----------------------~MERFRSMMN----GMGGQ--LGA~APGT0LIDNAET
NCU00823.1	TPPPASCGKLPIGAACSMLQPDA0MDRMRSLLG-GGLGMGGATAPGA0DNTN~LIDNSET
FG00781.1 	-----------------------~MERFRSLLGNGGMGLGGA-AHGT0DNTN~LIDNSET
AN4492.1  	-----------------MLSSAC~LYIFRLLGILCSAALKPETYYGQ0DTPN~LLDNSET
          	                   .. . :  :* :    . .:   :  *     . *:**:**

MG05858.1 	VYISSLALLKMLRHGRAGVPMEVMGLMLGEFVDDFTVRVVDVFAMPQSGTGVSVEAVDPV
NCU00823.1	VYISSLALLKMLRHGRAGVPMEVMGLMLGEFVDDFTVRVVDVFAMPQSGTGVSVEAVDPV
FG00781.1 	VYISSLALLKMLRHGRAGVPMEVMGLMLGEFVDDFTVKVMDVFAMPQSGTGVSVEAVDPV
AN4492.1  	VHISSLALLKMLRHGRAGVPMEVMGLMLGEFVDEYTVRVTDVFAMPQSGTGVSVEAVDPV
          	*:*******************************::**:* ********************

MG05858.1 	FQMKMMDMLRQTGR2PESVVGWYHSHPGFGCWLSSVDINTQQSFEQLTPRAVAVVVDPIQ
NCU00823.1	FQMNMMDMLRQTGR2PEAVVGWYHSHPGFGCWLSSVDINTQQSFEQLNSRAVAVVIDPIQ
FG00781.1 	FQTKMMDMLRQTGR2PESVVGWYHSHPGFGCWLSSVDINTQQSFEQLNPRAVAVVIDPIQ
AN4492.1  	FQTKMMDMLRQTGR2PEPVVGWYHSHPGFGCWLSSVDINTQQSFEQLTPRAVAVVVDPIQ
          	** :********** **.*****************************..******:****

MG05858.1 	SVKGKVVIDAFRLINPQSLMLGQEPRQSTSNLGHLNKPSIQALIHGLNRHYYSININYRK
NCU00823.1	SVKGKVVIDAFRLINPQSLMLGQEPRQTTSNLGHLNKPSIQALIHGLNRHYYSIGINYRK
FG00781.1 	SVKGKVVIDAFRLINPQLLMLGQEPRQSTSNLGHLNKPSIQALIHGLNRHYYSIGIDYRK
AN4492.1  	SVKGKVVIDAFRLIQPQTVVMGQEPRQTTSNLGHLNKPSIQALIHGLNRHYYSIGINYRK
          	**************:** :::******:**************************.*:***

MG05858.1 	TALEENMLMNLHKQVWTESLQMDDFRTQGQNNKERLDRLVSLSEGYEKRVKEETELTKDQ
NCU00823.1	TALEENMLMNLHKHVWTEALEMEDFRCEGSRTKERLDRLVSLADGYEKRVKEETELTKDQ
FG00781.1 	TALEENMLMNLHKHVWTEALEMNDFRHEGCKNKDRLQQLVTLADGYEKRVKEETELTKDQ
AN4492.1  	TGLEENMLMNLHKQVWTEALQMNDFHDECQHNVDRMKQLVNLAEGYEKRVKEETELTKDQ
          	*.***********:****:*:*:**: :  .. :*:.:**.*::****************

MG05858.1 	LKTRYVGKLDPKKH~LEDVGQQLIEDNIVAVSRQMIDKEAAVLEKKASAVGASGKGRSED
NCU00823.1	LKTRYVGKVDPKKH~LEDVGQQLIEDNIVAVTRQMIDKEATIAKKDTPAG---ANGTTHG
FG00781.1 	LKTRYVGKLDPKKH~LEDVGQELIEDNIVSVSRQMIDREATMPKRETPAG---SKGQVNG
AN4492.1  	LKTRYVGKVDPKKH1IEDVSQQLIEDNIVAVSRQMIDKEASVARQSNGAD---GKEPQNG
          	********:***** :***.*:*******:*:*****:**:: .:.  *    .:   ..

MG05858.1 	DQMDVEEDL-
NCU00823.1	DQMEVEEEQL
FG00781.1 	EEMDVEEEL-
AN4492.1  	VGMDVDEEL-
          	  *:*:*:
```
